# C9orf72-generated poly-GR and poly-PR do not directly interfere with nucleocytoplasmic transport

**DOI:** 10.1038/s41598-019-52035-6

**Published:** 2019-10-31

**Authors:** Joni Vanneste, Thomas Vercruysse, Steven Boeynaems, Adria Sicart, Philip Van Damme, Dirk Daelemans, Ludo Van Den Bosch

**Affiliations:** 1 KU Leuven - University of Leuven, Department of Neurosciences, Experimental Neurology and Leuven Brain Institute (LBI), Leuven, Belgium; 2 VIB, Center for Brain & Disease Research, Laboratory of Neurobiology, Leuven, Belgium; 3 KU Leuven Department of Microbiology, Immunology and Transplantation, Laboratory of Virology and Chemotherapy, Rega Institute for Medical Research, Leuven, Belgium; 4 Department of Genetics, Stanford University School of Medicine, Stanford, USA; 5 University Hospitals Leuven, Department of Neurology, Leuven, Belgium

**Keywords:** Amyotrophic lateral sclerosis, Amyotrophic lateral sclerosis

## Abstract

Repeat expansions in the *C9orf72* gene cause amyotrophic lateral sclerosis and frontotemporal dementia characterized by dipeptide-repeat protein (DPR) inclusions. The toxicity associated with two of these DPRs, poly-GR and poly-PR, has been associated with nucleocytoplasmic transport. To investigate the causal role of poly-GR or poly-PR on active nucleocytoplasmic transport, we measured nuclear import and export in poly-GR or poly-PR expressing Hela cells, neuronal-like SH-SY5Y cells and iPSC-derived motor neurons. Our data strongly indicate that poly-GR and poly-PR do not directly impede active nucleocytoplasmic transport.

## Introduction

Hexanucleotide (G_4_C_2_) repeat expansions in the first intron of the *C9orf72* gene are the most common genetic cause of amyotrophic lateral sclerosis (ALS) and frontotemporal dementia (FTD)^1^. Healthy individuals most often have 2 to 8 of these repeats, while C9orf72-patients can have up to hundreds or thousands^2,3^. C9orf72-patients show a unique pathology characterised by cytoplasmic inclusions containing dipeptide repeat proteins (DPRs)^4–6^. Five different DPRs arise through non-canonical translation of the sense and antisense repeat RNA, namely poly-GA (Glycine-Alanine), poly-GP (Glycine-Proline), poly-GR (Glycine-Arginine), poly-PA (Proline-Alanine) and poly-PR (Proline-Arginine)^4–7^. Although, DPRs are toxic in both cell culture and animal models, with the arginine containing poly-GR and poly-PR peptides as the most toxic ones (reviewed by Freibaum and Taylor, 2017^8^), the exact pathological mechanisms by which these DPRs contribute to neurodegeneration in C9orf72-ALS/FTD patients remains disputed.

We and others have previously reported, based on both yeast and *Drosophila* models, that the toxicity induced in mutant C9orf72 models can be modified by genetic or pharmacological manipulation of proteins involved in nucleocytoplasmic transport^9–12^ (reviewed by Yuva-Aydemin and colleagues, 2018^13^). In addition, changes in expression levels or cellular localization of nucleocytoplasmic transport proteins have been observed in mutant C9orf72-iPSC-derived motor neurons and *post mortem* tissue of C9orf72-patients^9,11,12,14,15^. Furthermore, a reduced import has been measured in C9orf72-iPSC-derived motor neurons^11,16^. These data argue for an important role of nucleocytoplasmic transport in the pathogenic mechanisms underlying C9orf72-ALS/FTD. However, there is currently no consensus on the mechanism(s) underlying the observed nucleocytoplasmic transport pathology. Interestingly, the poly-GR and poly-PR DPRs could be potential interactors of phenylalanine-glycine repeat-containing nucleoporines (FG Nups)^17^. FG Nups have a low sequence complexity^17^ and undergo phase separation into a dense polymer meshwork which constitute the nucleopore complex (NPC) permeability barrier^18^. This raises the intriguing possibility that poly-GR and poly-PR causally affect motor neuron health through disturbing nucleocytoplasmic transport directly. Therefore, the aim of this study was to measure the direct effect of several DPRs, including poly-GR and poly-PR, on active nucleocytoplasmic transport.

## Results

### Measuring active nucleocytoplasmic transport

**Figure 1:**
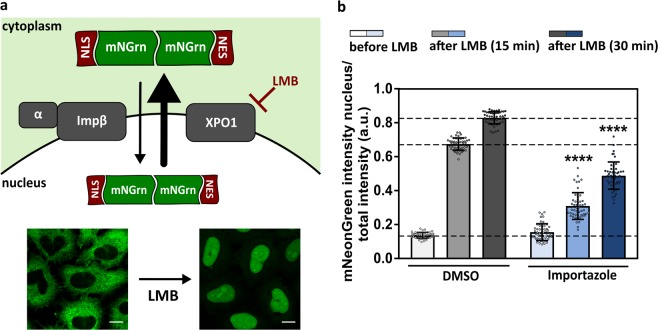
Active nucleocytoplasmic transport assay. (**a)** Hela Kyoto cells stably express the reporter NLS_SV40_-mNeonGreen2x-NES_pki_. The classical nuclear localization signal (NLS) is recognized by an importin α/β heterodimer (Impα/β), which results in the translocation of the reporter into the nucleus. The nuclear export signal (NES) of the reporter construct is recognized by exportin1 (XPO1), which is subsequently exported into the cytoplasm. Due to the strong nuclear export signal, the mNeonGreen-reporter is mainly cytoplasmic under control conditions. Addition of the XPO1-inhibitor leptomycin B (LMB) induces a shift in the localization of this reporter towards the nucleus, which allows us to measure nuclear import over time. Left image represents Hela Kyoto cells in control conditions. Right image represents Hela Kyoto cells after addition of leptomycin B. Scale bar = 10 µm. **(b)** The concentration of mNeonGreen in the nucleus was measured by fluorescent intensity before (as a measurement for XPO1-mediated export) and after (as a measurement for classical nuclear import) addition of leptomycin B. A significant import defect was induced by the importin-β inhibitor Importazole (time 15: p < 0.0001; time 30: p < 0.0001). Data are represented as mean ± SD, Mann-Whitney U-test, ****Denotes p < 0.0001. Dots represent means of one image with 5 cells per image; n = 20 from four independent biological replicates.

To measure active nucleocytoplasmic transport in intact cells, we made use of Hela Kyoto cells stably expressing the shuttling reporter NLS_SV40_-mNeonGreen2x-NES_pki_ (Fig. 1a). This mNeonGreen-construct, fused to both a classical nuclear localization signal (NLS) and an XPO1-associated nuclear export signal (NES), allows us to measure classical importinβ/α-mediated import and XPO1-mediated export in a quantitative matter. Notably, classical nuclear import is the most prevalent import pathway in the cell^19^ and its disturbance has been suggested to underlie cytoplasmic mislocalization of TAR DNA-binding protein 43 (TDP-43)^20^, which is a prominent hallmark of ALS and FTD^21^. In addition, the presence of two connected mNeonGreen proteins limits size-dependent passive transport across the NPC^22^, which allows us to primarily focus on active nucleocytoplasmic transport. Due to the strong nuclear export signal, the reporter is mainly cytoplasmic under control conditions (Fig. 1a). Inducing a shift in the localization of this reporter towards the nucleus, by blocking nuclear export using leptomycin B (LMB), allows us to measure nuclear import over time (Fig. 1a,b). As a control, we showed that the importin-β inhibitor Importazole^23^ significantly reduced import (p < 0.0001) (Fig. 1b).

### GR_20_ and PR_20_ poly-dipeptides do not block active nuclear import or export

**Figure 2:**
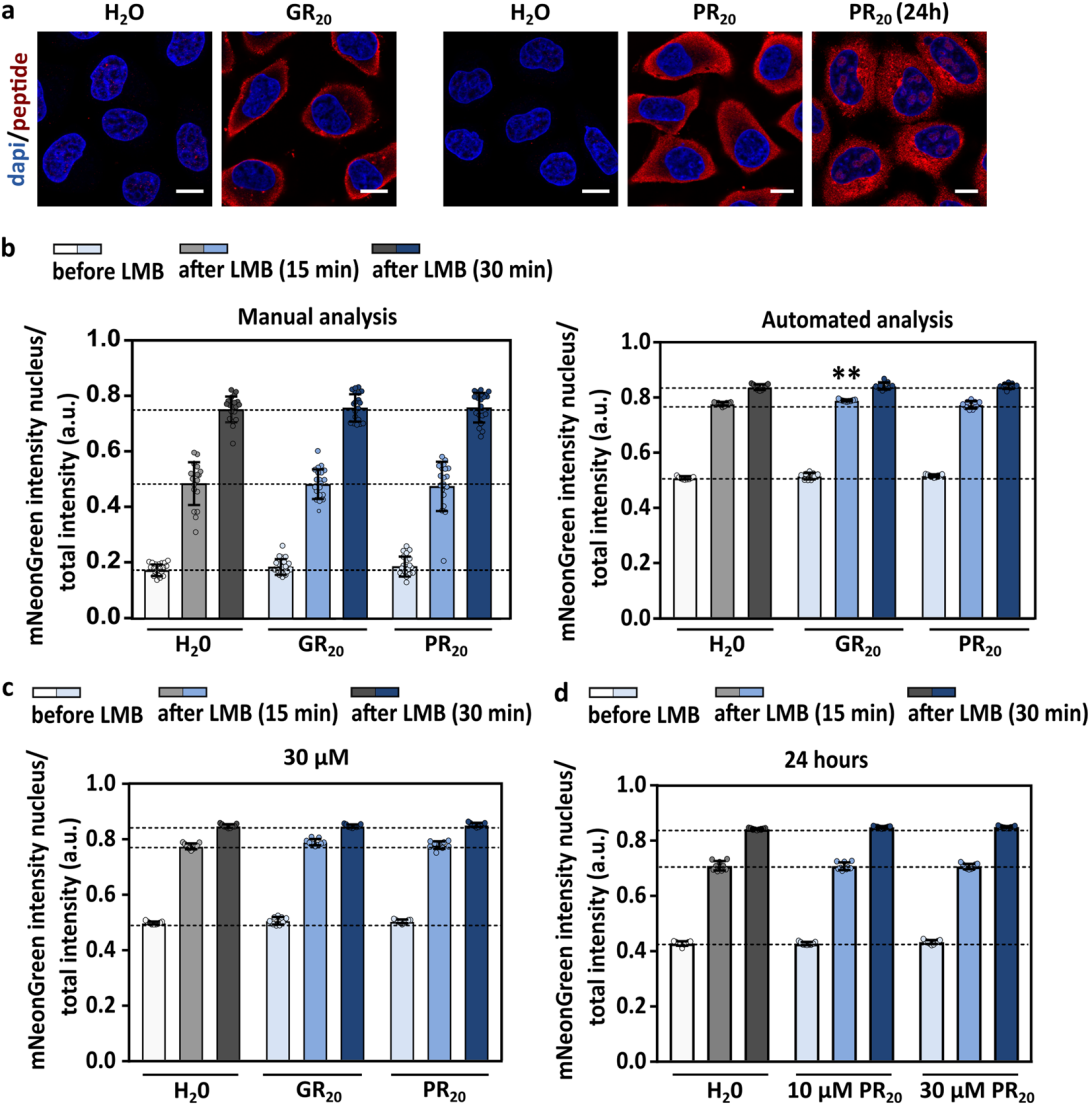
GR_20_ and PR_20_ poly-dipeptides do not block classical nuclear import and XPO1-related export. Nucleocytoplasmic transport was measured in the reporter Hela Kyoto cells in the presence of GR_20_ or PR_20_ peptides. **(a)** Cellular uptake of GR_20_ or PR_20_ was confirmed by immunocytochemistry, making use of antibodies directed against the peptides. Left two images were stained with an anti-poly-GR antibody (Millipore). Images on the right were stained with a homemade anti-poly-PR antibody. Scale bar = 10 µm. **(b)** Both manual (left graph) as automated (right graph) analyses were performed to measure active nucleocytoplasmic transport after two hours of incubation with 10 µM of GR_20_ or PR_20_. mNeonGreen fluorescent intensity was measured in the nucleus and cytoplasm, before and after addition of leptomycin B (LMB) (15 and 30 min). GR_20_ or PR_20_ peptides did not induce transport defects. A small but significant increase in import was observed for GR_20_ in the automated analysis (time 15: p = 0.0019). Neither a higher concentration (30 µM, automated analysis) **(c)** nor a longer incubation time (24 hours, automated analysis) **(d)** resulted in transport defects. Data are represented as mean ± SD, non-parametric one-way ANOVA followed by Dunn’s multiple comparison test, **Denotes p < 0.01. Manual analysis: dots represent means of one image with 5 cells per image; n = 19–20 from four independent experiments. Automated analysis: dots represent means of one well with 1499–4408 cells per well; n = 9 from three independent experiments.

In order to investigate the direct effect of poly-GR or poly-PR on active nucleocytoplasmic transport, we added 10 µM of peptides containing 20 repeats of either GR or PR (GR_20_ and PR_20_, respectively) to the Hela Kyoto reporter cell line. GR_20_ and PR_20_ are cell-penetrating peptides that are taken up immediately^24^, which we confirmed by immunocytochemistry (Fig. 2a). We did not observe an increased nuclear intensity of the reporter construct after two hours of incubation with GR_20_ and PR_20_ (Fig. 2b – before LMB). This suggests that the poly-dipeptides GR_20_ and PR_20_ do not obstruct XPO1-mediated export. Likewise, addition of LMB, which allows us to measure classical nuclear import, did not reveal a decreased nuclear import in the presence of both GR_20_ and PR_20_ (Fig. 2b – 15 and 30 min). Furthermore, no impeded transport was observed with a higher concentration of GR_20_ and PR_20_ (30 µM) or after a longer incubation with PR_20_ (24 hours; Fig. 2c,d).

Next to TDP-43 mislocalization, cytoplasmic mislocalization of the Fused in sarcoma (FUS) protein has recently been suggested as a common phenotype of ALS^25^. Therefore, we investigated whether poly-GR or poly-PR peptides could reduce FUS import through a direct interaction. Hela Kyoto cells expressing a shuttling reporter construct containing the same NLS as FUS were used to answer this question (supplementary Fig. S1A). FUS carries a non-classical proline-tyrosine NLS (PY-NLS) and is imported via transportin 1^26^. Hence, transfected cells expressing the transportin 1 inhibitor M9M^27^ had significantly reduced import of the reporter construct (p < 0.0001; Supplementary Fig. S1B). Similar to previous results, GR_20_ and PR_20_ did not induce disturbed transportin 1 import (Supplementary Fig. S1C).

### Intracellularly expressed poly-GR and poly-PR do not impede active transport

**Figure 3:**
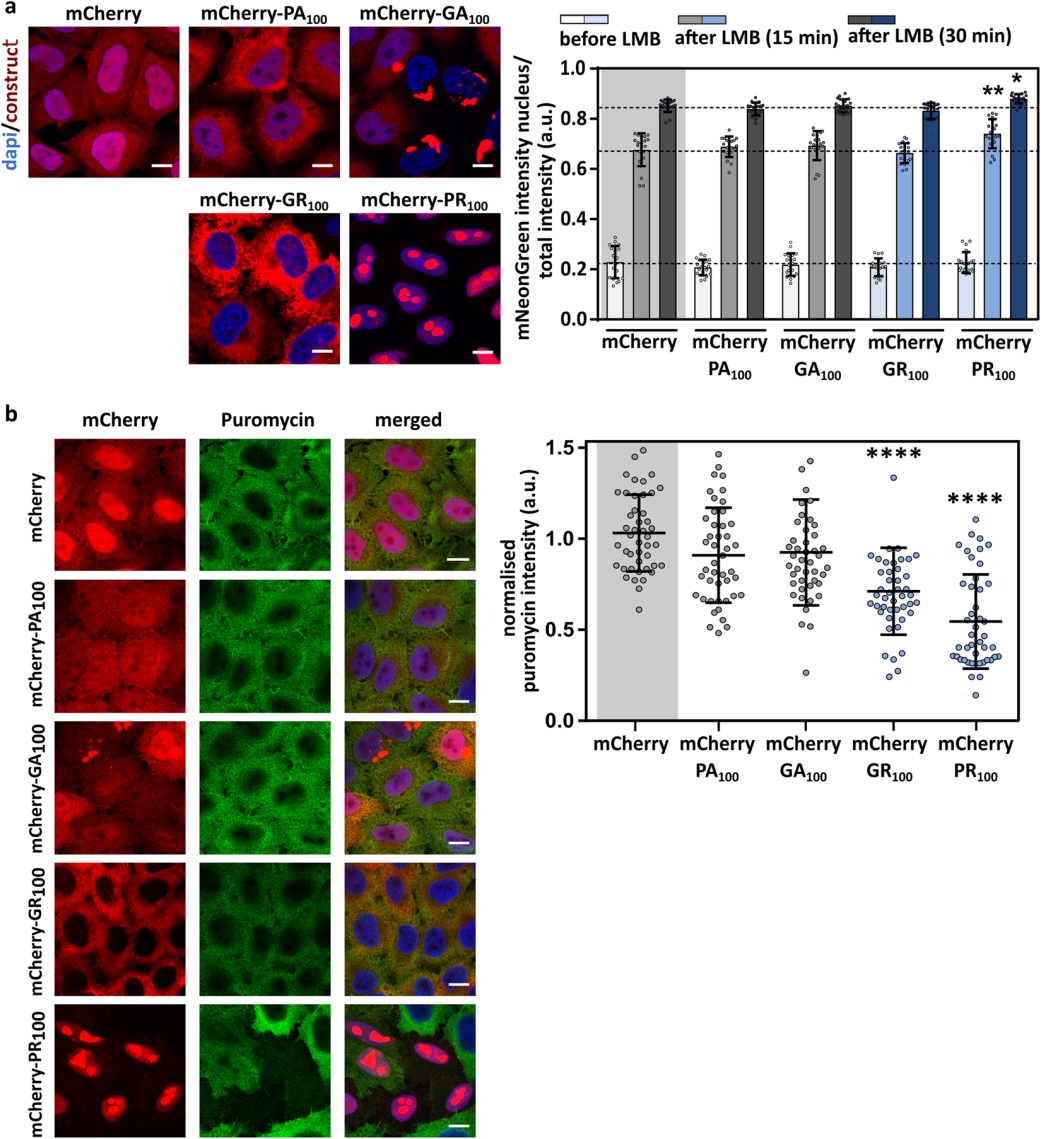
Intracellularly expressed poly-GR and poly-PR do not impede active transport in Hela Kyoto cells. (**a**) The left panel contains representative images of Hela Kyoto cells expressing the indicated constructs 96 hours after transduction. Scale bar = 10 µm. The right panel is the nuclear transport assay performed 96 hours after transduction of NLS_SV40_-mNeonGreen2x-NES_pki_-expressing cells with indicated constructs. No impeded transport was observed for any of the DPRs. A significant increased transport was observed in PR_100_-expressing cells (time 15: p = 0.0096; time 30: p = 0.0103). Data are represented as mean ± SD, non-parametric one-way ANOVA followed by Dunn’s multiple comparison test. Dots represent means of one image with 5 cells per image; n = 20 from four independent biological replicates. **(b)** To analyze the production of newly translated proteins, Hela Kyoto cells expressing indicated constructs were incubated with puromycin. Newly translated proteins, which are labeled with puromycin, were visualized by anti-puromycin antibody. Left panel represents double-immunostaining of Hela Kyoto cells for mCherry and puromycin. Right panel represents quantifications of newly translated proteins. Significant reduced protein  translation was observed in mCherry-GR_100_ and mCherry-PR_100_ expressing cells (GR: p < 0.0001; PR: p < 0.0001). Data are represented as mean ± SD, non-parametric one-way ANOVA followed by Dunn’s multiple comparison test, ****Denotes p < 0.0001. Dots represent means of one image; n = 45 from three independent experiments with 141–622 cells per experiment.

It has been shown that the length of DPRs has a clear effect, with the longer repeats being more toxic^12,28^. Therefore, we used lentiviral vectors (LVs) expressing 100 repeats of four different DPRs, namely mCherry-PA_100_, mCherry-GA_100_, mCherry-GR_100_ and mCherry-PR_100_. The Hela-reporter cell line was transduced 96 hours before measuring nucleocytoplasmic transport. As reported before^28^, poly-PA was diffusely present throughout the cell, poly-GA formed cytoplasmic inclusions, poly-GR was mainly cytoplasmic and poly-PR was mainly localized in the nucleoli (Fig. 3a). None of the intracellularly expressed DPRs affected nuclear export (Fig. 3a – before LMB) or import (Fig. 3a – 15 and 30 min). On the contrary, there was a small but significant (time 15: p = 0.007 – time 30: p = 0.0173) increase of import observed in mCherry-PR_100_ expressing cells.

To confirm that the obtained levels of poly-GR and poly-PR induced toxicity, we measured the effect on protein translation, as it was previously shown that poly-GR and poly-PR can induce toxicity by disturbing protein synthesis^24,29–32^. Indeed, reduced levels of protein translation was observed in poly-GR and poly-PR expressing cells (Fig. 3b,c).

A second nucleocytoplasmic transport assay confirmed that neither poly-GR nor poly-PR blocked nucleocytoplasmic transport (Supplementary Fig. S2). This assay consists of Hela Kyoto cells stably expressing the reporter NLS_c-myc_-GFP2x-NES_iKβ2_, which is mainly localized in the nucleus under control conditions (Supplementary Fig. S2A). Impeded import will shift the fluorescent signal to the cytoplasm, allowing us to measure disturbed nuclear import without blocking export. As a positive control, overexpression of the importin-β binding (IBB) domain of importin-α^33^ significantly reduced the nuclear intensity of the reporter (p < 0.0001 - Supplementary Fig. S2B). Despite the intracellular expression of the DPRs, no inhibitory effect on import was detected (Supplementary Fig. S2C).

### Intracellular expressed poly-GR and poly-PR do not impede active transport in neuronal like SH-SY5Y cells and iPSC-derived motor neurons

**Figure 4:**
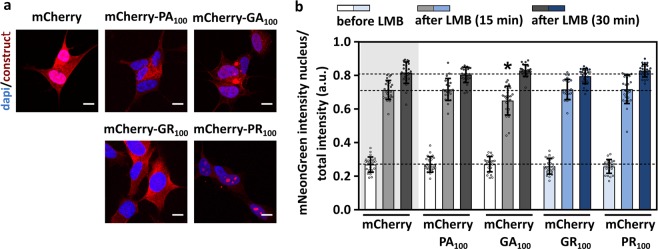
Intracellularly expressed poly-GR and poly-PR do not impede active transport in neuronal-like SH-SY5Y cells. (**a)** Representative images of SH-SY5Y cells expressing the indicated constructs 96 hours after transduction. Scale bar = 10 µm. **(b)** Nuclear transport assay performed 96 hours after co-transduction of SH-SY5Y cells with indicated constructs and the reporter construct NLS_SV40_-mNeonGreen2x-NES_pki_. Significant impeded transport was observed in GA_100_-expressing cells (time 15: p = 0.0488). Data are represented as mean ± SD, non-parametric one-way ANOVA followed by Dunn’s multiple comparison test, *Denotes p < 0.05. Dots represent means of one image with 5 cells per image; n = 24 from three independent biological replicates.

**Figure 5:**
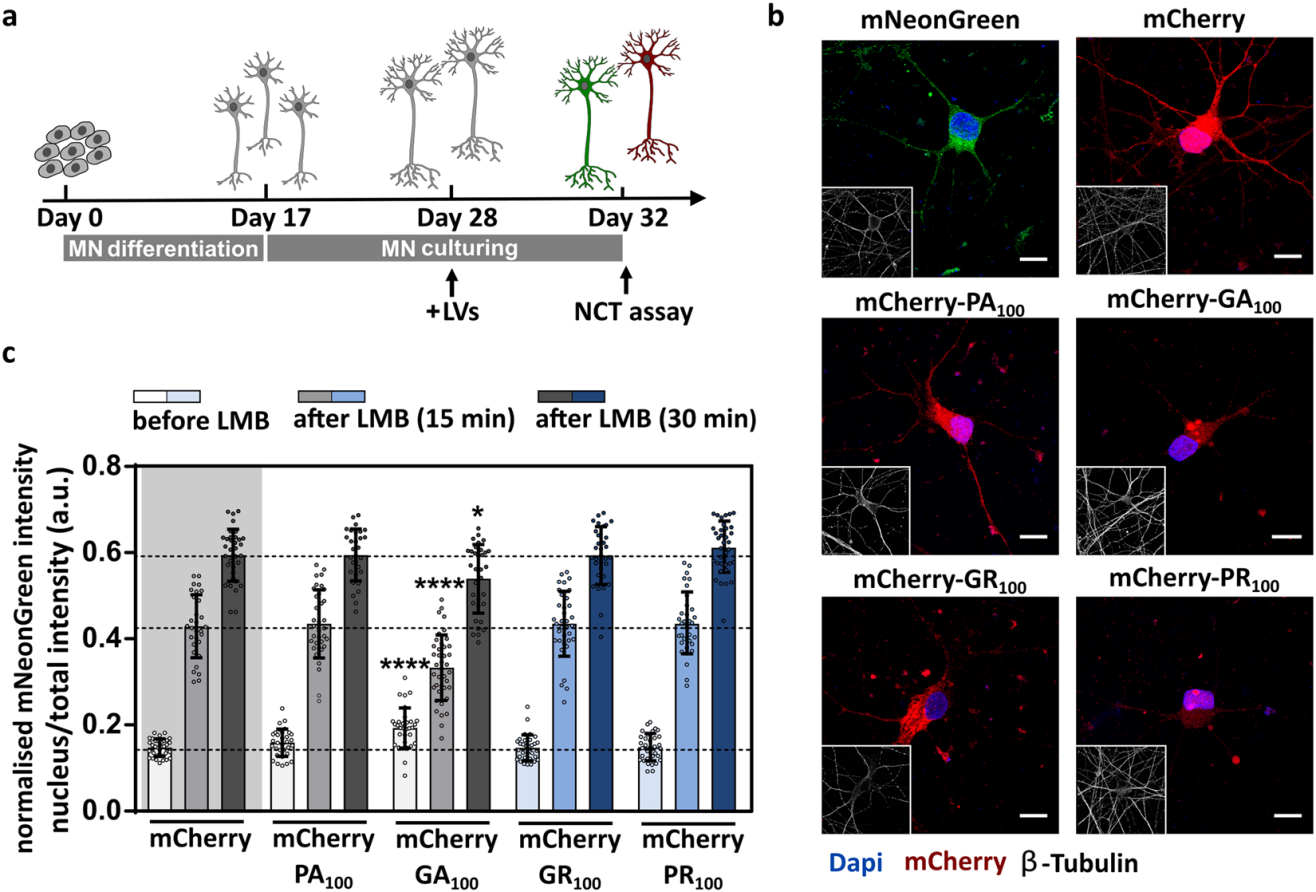
Intracellularly expressed poly-GR and poly-PR do not impede active transport in iPSC-derived motor neurons. (**a)** Healthy donor-derived iPSCs underwent a 17-day long differentiation into motor neurons (MNs) and were subsequently cultured up to 32 days. On day 28, motor neurons were co-transduced with LVs expressing a mCherry-DPR construct (PA_100_, GA_100_, GR_100_, PR_100_) or mCherry alone and a LV expressing NLS_SV40_-mNeonGreen2x-NES_pki_. Nucleocytoplasmic transport was measured on day 32. **(b)** Images represent 32-day old motor neurons expressing the indicated constructs 96 hours after transduction. Scale bar = 10 µm. **(c)** Nucleocytoplasmic transport measured at day 32. Significant disturbed transport observed for poly-GA expressing motor neurons (time 0: p < 0.0001; time 15: p < 0.0001; time 30: p = 0.0334). Data are represented as means ± SD, non-parametric one-way ANOVA followed by Dunn’s multiple comparison test. Dots represent means of one image with ± 5 cells per image; n = 30–40 from four independent biological replicates. Means ± SD. *Denotes p < 0.05; ****Denotes p < 0.0001.

As it has been shown that NPC proteins are cell-type specific^34^, we further investigated the effect of DPRs on nucleocytoplasmic transport in neuronal-like SH-SY5Y cells (Fig. 4) and in iPSC-derived motor neurons (Fig. 5). Healthy donor-derived iPSCs were differentiated into motor neurons as we published before^35^. The nucleocytoplasmic transport assay was performed 96 hours after co-transduction of SH-SY5Y cells (Fig. 4a) or motor neurons (Fig. 5a,b) with LVs expressing a mCherry-DPR construct as well as the NLS_SV40_-mNeonGreen2x-NES_pki_ reporter. We could confirm that poly-GR or poly-PR did not induce a blockade of nucleocytoplasmic transport (Figs 4b and 5c). Moreover, no disturbed transport was observed in poly-PA expressing cells (Figs 4b and 5c), which is in line with the non-toxic characteristics of poly-PA (reviewed by Freibaum and Taylor, 2017^8^). On the other hand, significant export (Fig. 5c - time 0: p < 0.0001) and import (Fig. 4b: p = 0.0488 and Fig. 5c - time 15 min: p < 0.0001 and 30 min: p = 0.0334) defects were observed in poly-GA expressing cells, as reported previously^36,37^.

## Discussion

To investigate the previously observed link between nucleocytoplasmic transport and mutant C9orf72^9–12,14,15,38^ in more detail, we measured the effect of poly-GR and poly-PR on active nucleocytoplasmic transport. Overall, our data do not support a directly induced impediment of nucleocytoplasmic transport by the most toxic DPRs, poly-GR or poly-PR.

Three alternative, not mutual exclusive, explanations for the observed association between mutant C9orf72 and nucleocytoplasmic transport are possible. First, it has been demonstrated that various cellular stresses can induce perturbation of conventional nucleocytoplasmic transport pathways and this through several mechanisms, including post-translational modifications and relocalization and degradation of transport factors (reviewed by Kose and Imamoto, 2014^39^). Interestingly, multiple mechanisms driving poly-GR/PR toxicity have been identified, such as reduced translation and mitochondrial stress (reviewed by Balendra and Isaacs, 2019^40^). This suggests that the previously observed interaction between nucleocytoplasmic transport and poly-GR or poly-PR-induced toxicity^9,10,12^ could be caused by an indirect stress-induced mechanism. For example, expression of poly-GR and poly-PR peptides can induce the formation^29,41^ and influence the composition of stress granules (SGs)^42^. These induced SGs can subsequently recruit nucleocytoplasmic transport factors, which results in nucleocytoplasmic transport deficits^43^. We rarely observed SG formation in poly-GR_100_ expressing cells and in less than 15% of poly-PR_100_ expressing cells (Supplementary Fig. S3A). The low number of SGs could explain why no transport defects were observed in contrast to the data published by Zhang and colleagues^43^. Indeed, when we only take mCherry-PR_100_ expressing Hela Kyoto cells that contain SGs into account, significant nucleocytoplasmic transport defects are observed (Supplementary Fig. S3B,C).

Second, a combination of other disease-related factors such as TDP-43 aggregates^14,15,44^, GA aggregates^36,37^, RNA foci^11^, cytoskeletal dysfunction^16^ and aging^45^ might explain the observed nucleocytoplasmic transport pathology in C9orf72-patients. We observed poly-GA induced nucleocytoplasmic transport deficits in SH-SY5Y cells and iPSC-derived motor neurons. Cell type-specific susceptibility factors might explain the difference with the results observed in Hela Kyoto cells. It is known that NPC proteins are cell-type specific^34^ and that the central nervous system is particular sensitive to nucleoporin defects^46^.

Third, nucleocytoplasmic transport proteins have non-transport related functions, including chaperone activity^47^. These alternative functions could explain some of the observed modifier effects of nucleocytoplasmic transport related proteins on C9orf72-induced toxicity, as they play a role in FUS and TDP-43 aggregation^48^.

Our findings are in clear contrast with previous data reporting a nucleocytoplasmic transport block induced by PR_20_ in non-neuronal systems^49^. This difference might be due to a different experimental set-up as well as to the use of different cell lines, as mainly *Xenopus laevis* oocytes, U2OS cells and digitonine-permeabilized Hela cells were used. As we did not observe blocked nucleocytoplasmic transport in intracellularly poly-GR_100_/PR_100_ expressing human motor neurons, we suggest that poly-GR and poly-PR do not drive neurotoxicity in C9orf72-ALS/FTD through a direct effect on nucleocytoplasmic transport. Still, we cannot exclude that age-dependent influences on the NPC and nucleocytoplasmic transport factors might play a role, as iPSC-derived neurons have been shown to be not yet fully mature^50^. Nevertheless, various pathological phenotypes have been observed in iPSC-derived neurons before^51^. However, it might also be possible that a life-long accumulation of poly-PR and poly-GR at the NPC is needed before problems occur.

In summary, poly-GR and poly-PR induced toxicity has previously been linked to nucleocytoplasmic transport. However, a better understanding of the underlying mechanisms is urgently needed to translate these findings in new therapeutic strategies. In this paper, we did not observe a directly induced obstruction of nucleocytoplasmic transport by poly-GR or poly-PR in Hela Kyoto cells, SH-SY5Y cells and human-derived motor neurons. This strongly suggests that a direct effect of poly-GR or poly-PR on nucleocytoplasmic transport is not the primary factor in the destructive pathway observed in C9orf72-patients. Our data rather support the hypothesis that indirect mechanisms underlie the previously observed modifying effect of nucleocytoplasmic transport factors on poly-GR or poly-PR induced toxicity.

## Material and Methods

### Cell culture

Hela Kyoto cells were grown in DMEM with high glucose and L-glutamine (ThermoFisher Scientific) supplemented with 10% fetal bovine serum (FBS) and gentamycin (20 μg/ml). SH-SY5Y cells (Sigma, 94030304) were maintained in DMEM-F12 medium (ThermoFisher Scientific) supplemented with 10% FBS and penicillin/streptavidin (100 μg/ml). Maintenance of human iPSCs (Takara Bio, Göteborg Sweden) and motor neuron differentiation was performed as described before^35^.

### Hela Kyoto reporter cell lines

The reporter construct NLS_SV40_-mNeonGreen2x-NES_pki2_ was integrated in the AAVS1 locus of Hela Kyoto cells under a CMV promotor using CRISPR-Cas9^52^. Hela Kyoto cells co-transfected with three plasmids were selected with 1 μg/ml puromycin and single-cell cloned. The following plasmids were used: a pcDNA3.1 plasmid containing the insert CMV-NLS_SV40_-mNeonGreen2x-NES_pki2_ followed by a puromycin-resistance gene (Puro^R^), a donor plasmid containing Cas9 plus a gRNA targeting the AAVS1 locus (GCCAGTCACCAATCCTGTCCCTAGTGG) and a donor plasmid containing gRNA to open the plasmid containing the mNeonGreen-reporter (GTACCCAAAAAGCGGGGGG).

A similar approach was used for the Hela Kyoto cell line expressing the reporter NES_pki2_-mNeonGreen2x-NLS_FUS_. A K to P mutation was induced in the NES_pki_–signal (MSLNELAL**P**LAGLDI) to obtain a nuclear signal under control conditions.

The reporter construct NLS_c-myc_-GFP2x-NES_iKβ2_ was integrated in the AAVS1 locus of Hela Kyoto cells under a CMV promotor using the CRISPPaint technique^53^. Hela Kyoto cells co-transfected with three plasmids were selected with 1 μg/ml puromycin and single-cell cloned. The following plasmids were used: a pcDNA3.1 plasmid containing the insert CMV-NLS_c-myc_-GFP2x-NES_iKβ2_ followed by Puro^R^, a donor plasmid containing Cas9 plus a gRNA targeting the AAVS1 locus (GTCACCAATCCTGTCCCTAGTGG) and a donor plasmid containing a gRNA (GCCAGTACCCAAAAAGCGGG) to cleave the plasmid containing the GFP-reporter.

### Lentiviral vector design

Flag-tagged DPR_100_ plasmids were a kind gift of Dr. Daisuke Ito (Department of Neurology, Keio University, Tokyo, Japan). These plasmids were used as a template to generate the LentiCrisprV2-Puro^R^-mCherry, LentiCrisprV2-Puro^R^-mCherry-PA_100_, LentiCrisprV2-Puro^R^-mCherry-GA_100_, LentiCrisprV2-Puro^R^-mCherry-GR_100_ and LentiCrisprV2-Puro^R^-mCherry-PR_100_ plasmids. Classical PCR reactions were used to clone the desired construct into the LentiCrisprV2-Puro^R^ plasmid, in which Cas9 was replaced. NLS_SV40_-mNeonGreen2x-NES_pki_ reporter construct were also cloned into a LentiCrisprV2-plasmid. Puromycin resistance gene was removed for the SUnSET assay. Constructs are expressed under an EF-1α-promotor. The sequence of all plasmids was verified by sequence analysis.

### Lentiviral vector production and addition

HEK293T cells were maintained in Dulbecco Modified Eagle Medium (DMEM; Gibco^TM^) with high glucose and L-glutamine supplemented plus 10% heat-inactivated fetal bovin serum (FBS) and gentamycin (20 μg/ml).

Production of lentiviral vectors was done as previously described^54^. Briefly, HEK293T cells were co-transfected with vectors psPAX2 (packaging vector), pMDG.2 (envelope vector) and one of the DPR-plasmids, using X-tremeGene9 transfection reagent (Roche). At 24 hours after transfection, the medium was changed to serum-free, high BSA growth medium (DMEM, 1% BSA, 100 μg/ml penicillin/streptomycin). Virus-containing medium was harvested 48 hours after transfection and stored at −80 °C.

Transduction of Hela Kyoto and SH-S5Y5 cells was carried out in the presence of 10 μg/ml polybrene 96 hours before analysing nucleocytoplasmic transport. Transduction of iPSC-derived motor neurons was carried out without polybrene at day 28 of the differentiation protocol and 96 hours before measuring nucleocytoplasmic transport.

### GR_20_ and PR_20_ poly-dipeptides

The GR_20_ and PR_20_ peptides were synthesized by Pepscan (Lelystad, The Netherlands). Peptides were dissolved in milli-Q water and stored at −80 °C. Hela Kyoto cells were treated with 10 μM or 30 µM of GR_20_ or PR_20_ for 2 hours (serum-free medium) or 24 hours before measuring nucleocytoplasmic transport. As a result of the short half-life of GR_20_ (20–30 min)^24^, this peptide was added once more after 1 hour.

### Nucleocytoplasmic transport assay

Nucleocytoplasmic transport assays were performed on Hela Kyoto cells stably expressing the report construct NLS_SV40_-mNeonGreen2x-NES_pki2_ and SH-SY5Y cells/iPSC-derived motor neurons constitutively expressing the reporter construct NLS_SV40_-mNeonGreen2x-NES_pki2_ after transduction. Cells were incubated with GR_20_/PR_20_ peptides (2 hours, 24 hours) or transduced with indicated constructs (96 hours) before fixation with 4% paraformaldehyde (PFA) in PBS, before (0 min) and after (indicated time points) addition of 45 nM leptomycin B (LMB; InvivoGen, Toulouse, France). As positive control, Hela Kyoto cells were treated for 2 hours with Importazole (50 μM; Selleckchem, Munich, Germany) before the addition of LMB.

Hela Kyoto cells containing the reporter construct NLS_c-myc_-GFP2x-NES_iKβ2_ were fixed with 4% PFA 96 hours after transduction with indicated constructs. As positive control, cells were transfected with Lipofectamine P3000 (Thermo Fisher Scientific) according to the instructions of the manufacturer with a plasmid expressing an importin β binding domain (pBiT2.1-EF1α-IBB-mRFP-NES_pki_) and fixed after 24 hours.

Hela Kyoto cells containing the reporter construct NES_pki2_-mNeonGreen2x-NLS_FUS_ were fixed with 4% PFA after incubation with GR_20_/PR_20_ peptides. As positive control, cells were transfected with Lipofectamine P3000 (Thermo Fisher Scientific) according to the instructions of the manufacturer with a plasmid expressing the transportin1 and 2 import inhibitor M9M^27^ (pBiT2.1-EF1α-Dmr-M9M) and fixed after 24 hours.

Cells were imaged with a Leica SP8 confocal microscope. The fluorescent intensity of reporters in the nucleus and cytoplasm was analyzed with ImageJ. A minimum of 25 cells (Hela Kyoto), 40 cells (SH-SY5Y, iPSC-derived motor neurons) per condition per experiment were measured. A minimum of 5 different images of minimum two different coverslips was used. Automated analysis were performed with the use of the CellInsight CX5 high content screening platform (Thermo Scientific). A minimum of 5600 cells from three different wells per experiment were measured.

### SUnSET assay

To examine the production of newly translated proteins, a SUnSET assay was performed as previously described^55^. In short, Hela Kyoto cells were grown in 24-well plates on glass coverslips and transduced with indicated constructs. After 96 hours, cells were incubated with fresh medium containing 1 μg/ml puromycin and fixed after 1 hour to perform immunostaining. Cells were imaged with a Leica SP8 confocal microscope and analyzed automatically with Cellprofiler. A total of 15 images from three different coverslips were used per experiment. 141–622 cells were measured per experiment. Data from three independent experiments were pooled.

### Immunofluorescence

Cells plated on coverslips were fixed in 4% paraformaldehyde for 20 min at room temperature and were washed with PBS. Permeabilization and blocking was done using PBS containing 0.1% Triton X-100 (Acros Organics) and 5% donkey serum (Sigma) for 1 hour. Cells were incubated overnight at 4 °C in blocking buffer (2% donkey serum) containing the different primary antibodies against poly-GR (1/500, EMB Millipore, MABN778), poly-PR (homemade with PR15 as antigen, rabbit, affinity purification), β-Tubuline (1/500; Abcam; ab7751), anti-G3BP (1/500; Abcam; ab56574) or anti-puromycin (1/1000, Millipore, MABE342). After washing with PBS, cells were incubated with corresponding secondary antibodies (1/2500, Invitrogen) for 1 hour at room temperature. Nuclei were stained using NucBlue Live Cell Stain reagent (Invitrogen). Fluorescent images were taken with Leica SP8 confocal microscope.

### Sodium arsenite treatment

Hela Kyoto cells were treated with 0.5 mM of sodium arsenite for 1 hour before fixation with 4% paraformaldehyde.

### Statistical analysis

Data of minimum three biological independent experiments were pooled in all analyses, of which each experiment contributed the same n. The mean of one image (manual analysis or Cellprofiler) or well (automatic analysis with CellInsight CX5 high content screening platform) was seen as one data point. Statistical analyses were performed on the log transformations for the nucleocytoplasmic transport assay. D’Agostino-Pearson omnibus normality test was used to test data for normality. Parametric tests were used on normally distributed data. Non-parametric tests were used on non-normally distributed data. Mann-Whitney U-test was used to determine statistical differences in the Importazole experiment. (Non)-parametric one-way ANOVA followed by Dunn’s multiple comparison/Dunnett’s multiple comparison test was used for the other experiments to determine statistical differences between groups. Data values represent mean ± SD. Graphpad Prism version 7 was used to perform statistical analyses.

## Supplementary information

Supplementary information

## Data Availability

All data generated or analysed during this study are included in this published article (and its Supplementary Information files) and are available from the corresponding author on reasonable request.
