# Supplementary material for: C9orf72-generated poly-GR and poly-PR do not directly interfere with nucleocytoplasmic transport
Source: Sci Rep. 2019 Oct 31;9:15728. doi: 10.1038/s41598-019-52035-6 (PMC6823349; doi:10.1038/s41598-019-52035-6)
Supplement: Supplementary file 1 — Supplementary information [file 41598_2019_52035_MOESM1_ESM.pdf]

C9orf72-generated poly-GR and poly-PR do not directly interfere with nucleocytoplasmic transport

Joni Vanneste[1,2], Thomas Vercruysse[3], Steven Boeynaems[4], Adria Sicart[1,2], Philip Van Damme[1,2,5], Dirk Daelemans[3] and Ludo Van Den Bosch[1,2 *]

1. KU Leuven - University of Leuven, Department of Neurosciences, Experimental Neurology and Leuven Brain Institute (LBI), Leuven, Belgium

2. VIB, Center for Brain & Disease Research, Laboratory of Neurobiology, Leuven, Belgium

3. KU Leuven Department of Microbiology, Immunology and transplantation, Laboratory of Virology and Chemotherapy, Rega Institute for Medical Research, Leuven, Belgium

4. Department of Genetics, Stanford University School of Medicine, Stanford, USA.

5. University Hospitals Leuven, Department of Neurology, Leuven, Belgium

* Correspondence and requests for materials should be addressed to L.V.D.B. (email: Ludo.Vandenbosch@kuleuven.vib.be)

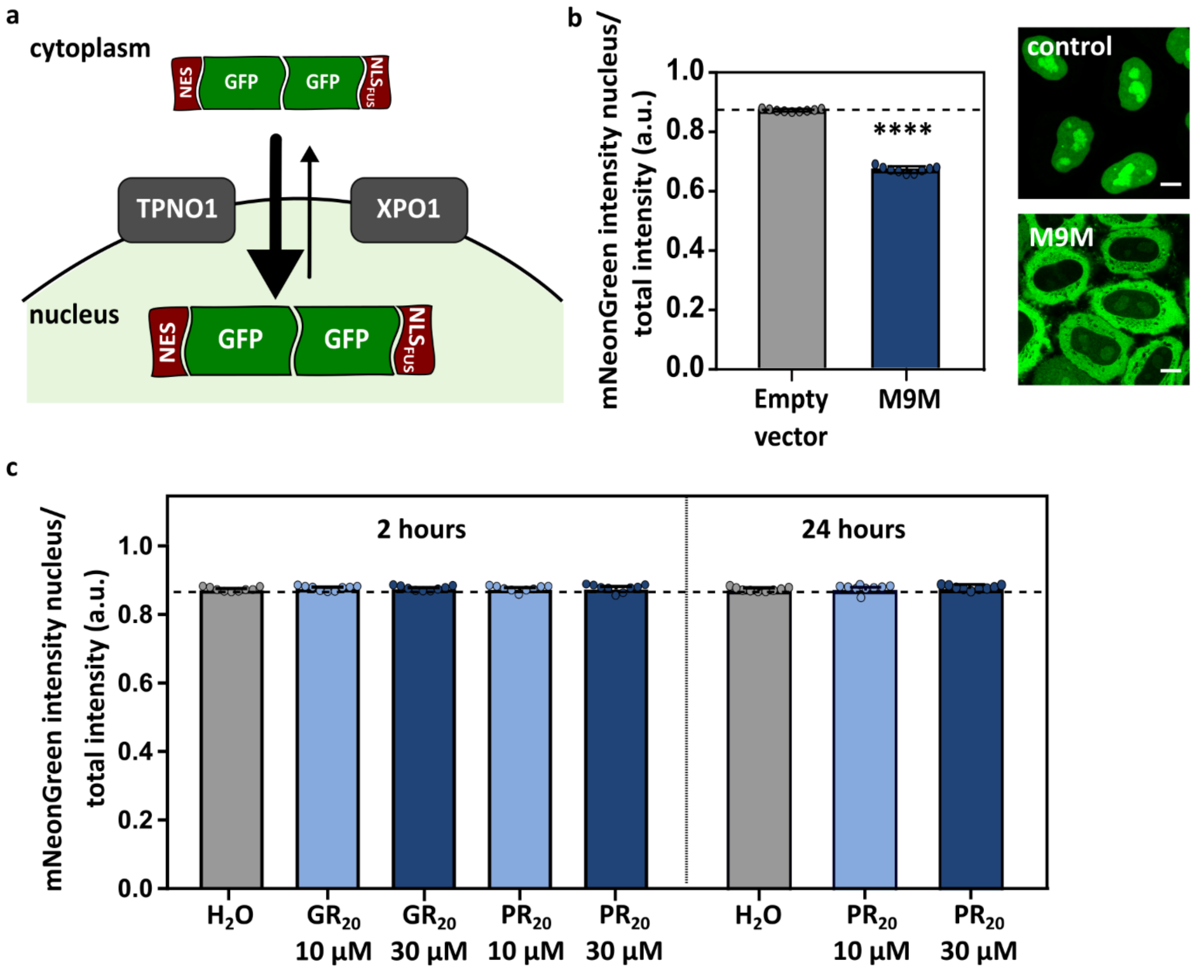

Supplementary Figure 1. GR_{20} and PR_{20} poly-dipeptides do not induce decreased FUS-related import. **(a)** Hela Kyoto cells stably expressing the shuttling reporter NES_{pki}-mNeonGreen2x-NLS_{FUS}. The FUS NLS is recognized by transportin 1 (TPNO1), which results in the translocation of the reporter into the nucleus. The nuclear export signal (NES) of the reporter construct is recognized by exportin1 (XPO1). Due to the strong NLS the mNeonGreen-reporter is mainly in the nucleus under control conditions. **(b)** Import was significantly reduced in cells transfected with plasmid expressing the transportin 1 inhibitor M9M ($p < 0.0001$; mix of tranfected and untransfected cells were measured, which diminishes the effect). Upper image represents control conditions. Lower image represents Hela Kyoto cells expressing the inhibitor M9M. Scale bar = 10 µm. **(c)** Nuclear intensity was automatically analyzed after incubation with GR_{20} or PR_{20} at indicated time points and concentrations. No TPNO1-import defects were observed. Data are represented as mean ± SD, b: unpaired t-test c: (non)-parametric one-way ANOVA followed by Dunn's/Dunnett's multiple comparison test, **** denotes p < 0001. Dots

represent means of well image with 1368 - 8567 cells per image; n = 9 from three independent biological replicates.

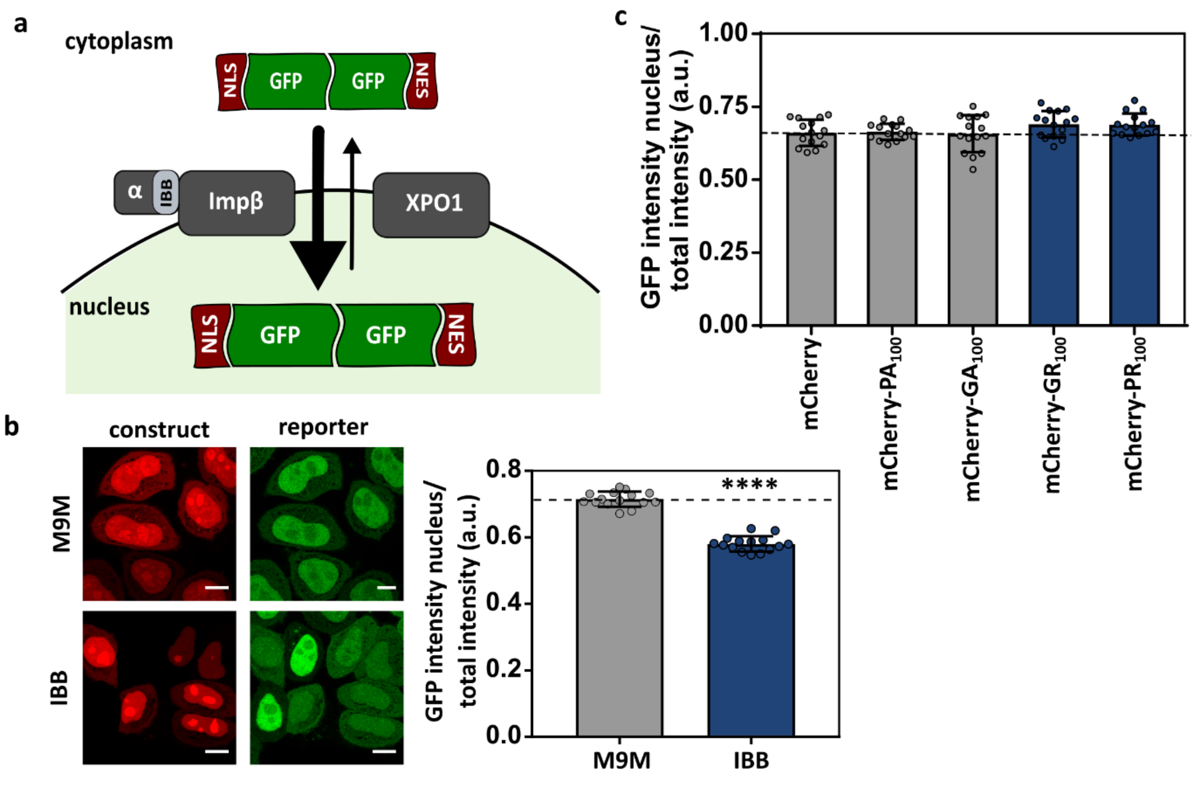

Supplementary Figure 2. Intracellularly expressed poly-GR and poly-PR do not block active import. **(a)** Hela Kyoto cells stably expressing the shuttling reporter construct $NLS_{c\text{-}myc}$-GFP2x-$NES_{iK\beta2}$. Importin-α (Impα) is an adaptor protein binding the classical NLS of the reporter and linking the latter to importin-β (Impβ) through its N-terminal IBB domain. The NES of the reporter construct is recognized by exportin1 (XPO1). GFP is mainly localized in the nucleus under control conditions due to the strong import signal. **(b)** Graph represents significant reduced import induced by overexpression of the import β binding (IBB) domain of importin α ($p < 0.0001$). Overexpression of the IBB domain promotes the dissociation of importin-β and, as such, results in decreased nuclear import. Upper images represent cells expressing the transport inhibitor M9M as a negative control. Lower images represents cell expressing the IBB domain as a positive control. Scale bar = 10 µm. **(c)** Nuclear intensity was manually analyzed 96 hours after transduction with the indicated constructs. No import defects were observed. Data are represented as mean ± SD, b: unpaired t-test; c: parametric one-way ANOVA followed by Dunnett's multiple comparison test. **** denotes $p < 0.0001$. Dots represent means of one image with 5 cells per image; n = 15 from three independent biological replicates.

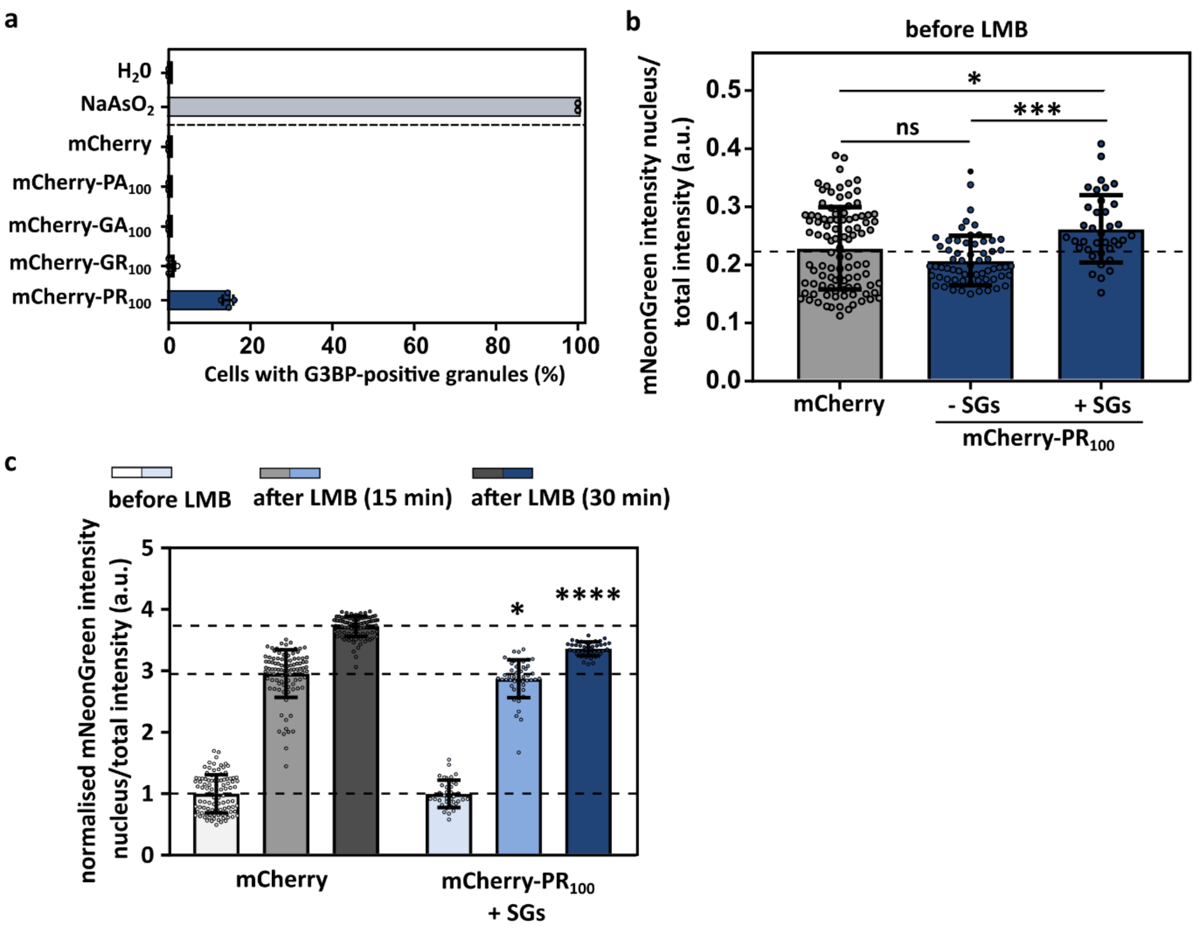

Supplementary figure 3. Nucleocytoplasmic transport deficits in mCherry-PR$_{100}$ expressing cells that contain stress granules. (a) Percentage of Hela Kyoto cells containing G3BP-positive stress granules manually counted 96 hours after transduction with indicated constructs or after treatment with H_2O or 0.5 mM sodium arsenite ($NaAsO_2$). Each dot represents the average of one experiment containing 60-152 cells with n = 3-4. G3BP-posive cells were rarely observed in mCherry-GR$_{100}$ expressing cells (0.51 %) and in less than 15 % of mCherry-PR$_{100}$ expressing cells (14.50 %). **(b)** mCherry-PR$_{100}$ expressing Hela Kyoto cells were categorized into two groups based on the presence of G3BP-positive stress granules (SGs). A significant higher nuclear intensity of the NLS$_{SV40}$-mNeonGreen2x-NES$_{pki}$ reporter was observed in poly-PR expressing cells containing stress granules compared to mCherry-expressing cells ($p = 0.0105$) and mCherry-PR$_{100}$ cells without SGs ($p = 0.0002$), suggesting a decreased nuclear export. **(c)** To account for the significant difference before the addition of leptomycin B (LMB), data were normalized to time 0. Significant reduced import was measured in poly-PR expressing cells containing stress granules (time 15: $p = 0.0149$; time 30: $p < 0.0001$). Data are represented as mean ± SD, (b) non-

parametric one-way ANOVA followed by Dunn's multiple comparison test, (c) Mann-Whitney u test. Dots represent individual cells pooled from four independent experiments; n = 36 -100. * denotes $p < 0.05$. *** denotes $p < 0.005$, **** denotes $p < 0.0001$.
